# An Electrospun Porous CuBi_2_O_4_ Nanofiber Photocathode for Efficient Solar Water Splitting

**DOI:** 10.3390/polym13193341

**Published:** 2021-09-29

**Authors:** Xiuhua Yuan, Yeling Liu, Hui Yuan, Bingxin Liu, Tianyu Guo, Huawei Zhou, Xia Li

**Affiliations:** 1 School of Mechanical and Automotive Engineering, Liaocheng University, Liaocheng 252000, China; yuanxiuhua@lcu.edu.cn; 2 Department of Chemistry, Liaocheng University, Liaocheng 252000, China; 1910120301@stu.lcu.edu.cn (Y.L.); 2010120316@stu.lcu.edu.cn (H.Y.); 2018204269@stu.lcu.edu.cn (B.L.); zhouhuawei@lcu.edu.cn (H.Z.); 3 Department of Art and Science, University of Vermont, Burlington, VT 05405, USA; tguo18@jh.edu

**Keywords:** electrospinning, CuBi_2_O_4_ nanofiber, photocathode, water splitting

## Abstract

While the CuBi_2_O_4_-based photocathode has emerged as an ideal candidate for photoelectrochemical water splitting, it is still far from its theoretical values due to poor charge carrier transport, poor electron–hole separation, and instability caused by self-photoelectric-corrosion with electrolytes. Establishing synthesis methods to produce a CuBi_2_O_4_ photocathode with sufficient cocatalyst sites would be highly beneficial for water splitting. Here, the platinum-enriched porous CuBi_2_O_4_ nanofiber (CuBi_2_O_4_/Pt) with uniform coverage and high surface area was prepared as a photocathode through an electrospinning and electrodeposition process for water splitting. The prepared photocathode material was composed of a CuBi_2_O_4_ nanofiber array, which has a freestanding porous structure, and the Pt nanoparticle is firmly embedded on the rough surface. The highly porous nanofiber structures allow the cocatalyst (Pt) better alignment on the surface of CuBi_2_O_4_, which can effectively suppress the electron–hole recombination at the electrolyte interface. The as-fabricated CuBi_2_O_4_ nanofiber has a tetragonal crystal structure, and its band gap was determined to be 1.8 eV. The self-supporting porous structure and electrocatalytic activity of Pt can effectively promote the separation of electron–hole pairs, thus obtaining high photocurrent density (0.21 mA/cm^2^ at 0.6 V vs. RHE) and incident photon-to-current conversion efficiency (IPCE, 4% at 380 nm). This work shows a new view for integrating an amount of Pt nanoparticles with CuBi_2_O_4_ nanofibers and demonstrates the synergistic effect of cocatalysts for future solar water splitting.

## 1. Introduction

It is imperative to find sustainable alternative energy to cope with humankind’s energy source crisis [1,2]. Photoelectrochemical water splitting for hydrogen under solar irradiation is seen as the ultimate way to solve the energy crisis [3,4,5,6]. A critical challenge for photoelectrochemical water splitting is the low conversion efficiency suffering from poor charge carrier transport and poor electron–hole separation. In order to improve the efficiency of water splitting, it is necessary to explore a new type of photoelectric material with the best band gap and photocurrent starting potential [7,8,9,10]. In this regard, copper-based oxides-based photocathodes with natural p-type conductivity are a very good choice for their high photocurrents [10,11,12]. Nevertheless, the Cu 3d character in the conduction band of Cu_2_O will lead to photoelectron-induced self-reduction and poor operational stability [10,11]. Therefore, it is urgent to develop a new type of copper-based metal oxide photocathode, so that the Cu_2_O conduction band is sheared, and the photogenerated electrons are directed to the redox stable metal orbitals.

Substantial studies have revealed CuBi_2_O_4_, a multinary p-type metal oxide semiconductor that alloys Cu_2_O with Bi oxide, and its ternary alloy structure allows the photogenerated electrons to be directed toward redox stable metal orbitals [12]. Such CuBi_2_O_4_ possesses a sufficiently narrow direct bandgap and exceptionally positive photocurrent onset potential (>1.0 V vs. RHE), thus improving solar energy utilization [13,14,15,16]. For example, a powder-type CuBi_2_O_4_ photocatalyst can be realized by hypoxic calcination [17], hydrothermal synthesis [18], and the sol−gel method [19]. Although the photocathode prepared by powder-type CuBi_2_O_4_ has various desirable properties, it has not achieved a high photoelectric conversion efficiency. Photocathode corrosion often occurs during oxygen reduction due to the poor transport property of the carrier (~1.2 × 10^−3^ cm^2^/Vs) [14]. Therefore, substantial improvements in activity and stability are greatly needed.

Recently, the coupling of film-type CuBi_2_O_4_ with different noble metal decorative materials has attracted widespread attention due to its synergistic effect, which can increase photoelectrochemical activity [20]. Such CuBi_2_O_4_ film can be realized by hydrothermal synthesis, chemical bath deposition [21], and a template-directed method [22]. For example, Xu et al. demonstrated Au coating film-type CuBi_2_O_4_ photocathodes with high photoelectrochemical activity through coupling p-type doping with Au and gradient Cu-vacancy doping [23,24]. Cao et al. fabricated CuBi_2_O_4_ film decorated with Pt nanoparticles using atomic layer deposition and indicated an attractive p-type material in water splitting without concern for the corrosion problem in aqueous electrolytes [16]. Park et al. reported a CuO|CuBi_2_O_4_ film coated with Pt layers for water splitting and showed more than double the photoactivity compared to the corresponding monolayer photocathode [25]. The photoelectrochemical properties of such polycrystalline thin films can vary significantly, which mainly depend on their morphological details (such as uniform coverage, surface area, and the size and number of cocatalysts) [26,27,28,29]. Although the thin-film CuBi_2_O_4_ photocathode system has a high application efficiency, its application scope is limited, especially in dense thin films, which often suffer from the recombination of electron–hole pairs [20]. Therefore, designing a simple and effective synthesis method to obtain a stable extensible film-type CuBi_2_O_4_ photoelectrode with sufficient active sites will be very conducive to improving water splitting.

Electrospinning provides a simple and scalable synthesis method to fabricate one-dimensional nanomaterial [30] and has been proven particularly useful in the field of photocatalysis [31,32,33]. While electrospinning has been used to fabricate CuO nanofibers [34] and BiVO_4_ nanotubes [35] for solar water splitting, no studies have been reported on fabricating CuBi_2_O_4_ nanofibers. Here, the novel platinum-enriched porous CuBi_2_O_4_ nanofiber (CuBi_2_O_4_/Pt) with uniform coverage and high surface area was prepared as a photocathode through an electrospinning and electrodeposition process for efficient water splitting. The prepared photocathode material was composed of a CuBi_2_O_4_ nanofiber array, which has a freestanding porous structure, and the Pt nanoparticle was firmly embedded on the rough surface. The porous nanofiber structure makes the cocatalyst (Pt) better arranged on the CuBi_2_O_4_ surface, which effectively prevents the electron–hole pair recombination at the electrolyte interface. The as-fabricated CuBi_2_O_4_ nanofiber has a tetragonal crystal structure, and its band gap was determined to be 1.8 eV. The self-supporting porous structure and cocatalytic activity of Pt can effectively promote the separation of electron–hole pairs, resulting in high photocurrent density (0.21 mA/cm^2^ at 0.6 V vs. RHE) and IPCE (4% at 380 nm). This study provides a new idea for the integration of Pt nanoparticles and CuBi_2_O_4_ nanofibers and provides a synergistic catalyst for future solar water splitting.

## 2. Materials and Methods

**Figure 1:**
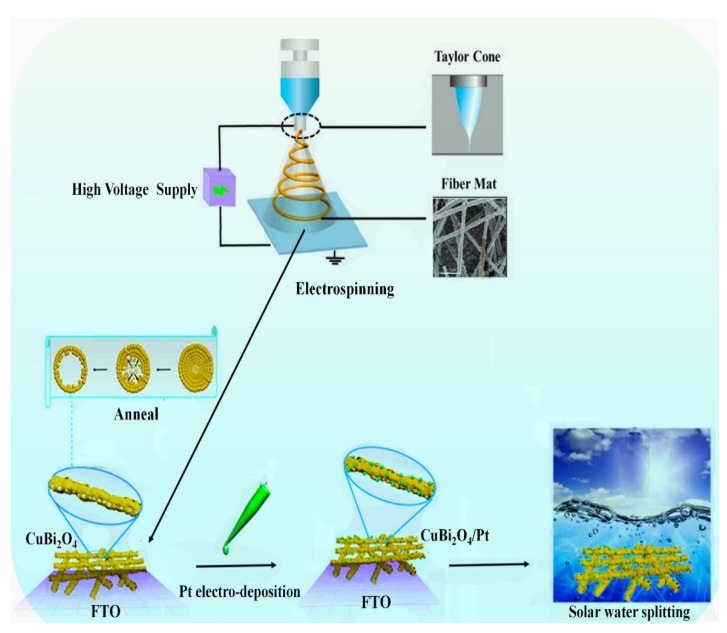
Schematic illustration of the CuBi_2_O_4_/Pt nanofiber fabrication process.

The CuBi_2_O_4_/Pt nanofiber film was synthesized via a three-step process: electrospinning, annealing, and deposition, which is shown in Figure 1.

### 2.1. Materials

The polyvinylpyrrolidone (PVP, K90, Mw = 1,300,000) and chloroplatinic acid (H_2_PtCl_6_·6H_2_O) were from Aladdin, Shanghai, China. The bismuth nitrate pentahydrate (Bi(NO_3_)_3_·5H_2_O), cupric nitrate (Cu(NO_3_)_2_·3H_2_O), N,N-Dimethylformamide (DMF), and acetic acid (CH_3_COOH) were obtained from J&K Chemical Ltd., B, Beijing, China. All materials were of analytical grade without further purification.

### 2.2. Preparation of Porous CuBi_2_O_4_/Pt Nanofiber Film

The synthesis of the precursor solution followed two steps: first of all, the Bi(NO_3_)_3_·5H_2_O and Cu(NO_3_)_2_·3H_2_O were added to a mixture of acetic acid and DMF and stirred 1 h to ensure dissolution; then the PVP was added to the above mixture and stirred 10 h to form the homogeneous precursor (Figure S1a, Supplementary Materials). The electrospinning was carried out with a self-made apparatus [35], which was composed of a plastic syringe, a high voltage supply, and a plate collector. The homogeneous precursor was injected into the syringe with a stainless steel needle (diameter = 0.5 mm). The FTO glass (OPV-FTO-22-07, 2.5 × 3 cm^2^) was pasted on the counter plate to collect nanofibers. The electrospinning was performed at a distance of 20 cm between the tip of the steel needle and the plate collector, at a high voltage of 20 kV, at an injection rate of 0.1 mL/h, and with an air humidity of 40%. After electrospinning for 25 min, the films collected on the FTO glass (Figure S1b, Supplementary Materials) were dried at 100 °C for 5 h. Based on the thermogravimetry curve (Figure S2, Supplementary Materials), the nanofiber film on FTO was annealed at 520 °C for 1 h and naturally cooled down to ambient temperature. Finally, the photocathode of CuBi_2_O_4_ nanofibers (Figure S1c, Supplementary Materials) was successfully obtained.

The Pt nanoparticles were loaded onto the CuBi_2_O_4_ nanofibers by electrodeposition, as the PtCl_6_^2+^ can be reduced to Pt nanoparticles at low potential. A three-electrode system was employed with an as-prepared nanofiber on FTO glass (working electrode), an Ag/AgCl reference electrode, and a platinum counter electrode. The electrolyte was 0.1 mM H_2_PtCl_6_•6H_2_O in 0.1 M potassium borate buffer (pH = 7.0). The electrodeposition was carried out using an electrochemical workstation (Zahner Zennium) at −0.20 V versus Ag/AgCl for 1 min.

### 2.3. Physical Characterization

In order to investigate the nanofiber, its morphology was measured by a scanning electron microscope (SEM, Zeiss Merlin), an energy-dispersive X-ray spectroscope (EDS), and a transmission electron microscope (TEM, JEM-2100). Its chemical element and crystallinity were characterized by X-ray photoelectron spectroscopy (XPS, ESCALAB Xi +) and X-ray diffraction (XRD, Bruker Smart-1000CCD diffractometer), respectively. The surface area of the CuBi_2_O_4_ nanofiber was measured by the Brunauer–Emmett–Teller (BET, Micromeritics ASAP2460, Norcross, GA, USA). The UV-visible diffuse reflectance spectrum was characterized by a UV-vis spectrophotometer (PE lambda 750) with an integrated sphere attachment.

The photocatalytic H_2_ was carried out using the CEL-PAEM-D8 photocatalytic activity evaluation system, which consisted of a gas chromatograph (AgilentTechnologies GC-7890B) and a 300 W Xe lamp (MicroSolar 300, Perfect Light). The circulation water of 25 °C was applied to maintain the reaction temperature of the solution. The photoelectrochemical experiments on the CuBi_2_O_4_/Pt nanofiber photocathode were performed on the Zahner electrochemical workstation in a three-electrode cell (the CuBi_2_O_4_/Pt nanofibers on FTO, Ag/AgCl reference electrode, and a platinum counter electrode, respectively). For photocurrent measurements, the electrolyte was 0.2 M PBS (pH 7.0). The Xe lamp (CEL-HXF300-T3, P = 100 mW/cm^2^, AM1.5) was used as the illumination source. The light intensity was adjusted by a calibrated photodetector. For incident photon-to-current efficiency (IPCE) measurements, the CIMPS TLS03 model (Zahner tunable light source system) was employed for monochromatic light excitation. The chopped photocurrent–voltammetry measurement was carried out with a scan speed of 10 mV/s and a chopped light time of 8 s.

## 3. Results

### 3.1. Morphology and Structure of Nanofibers

**Figure 2:**
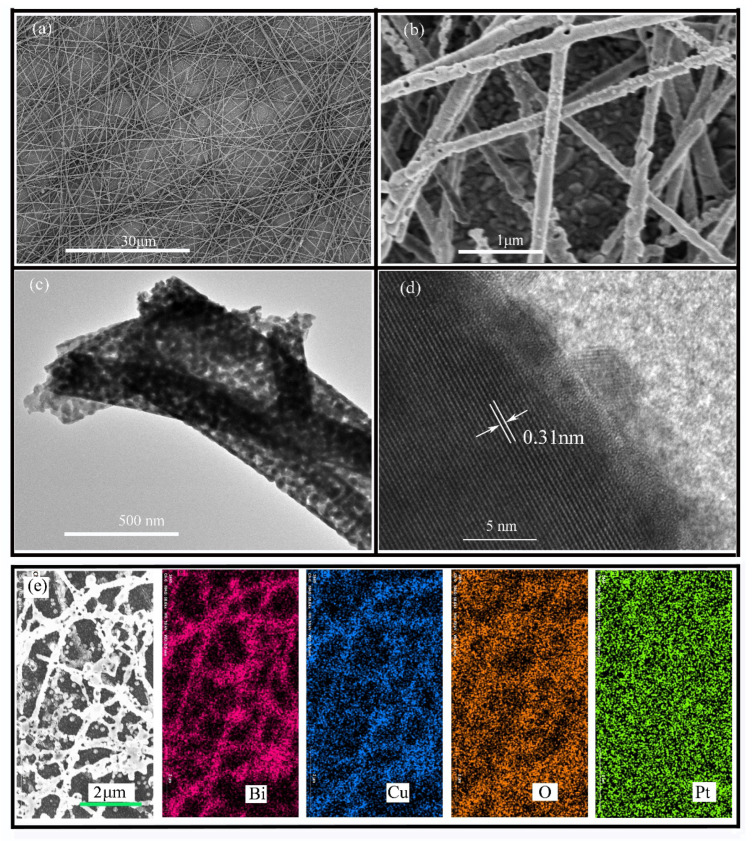
The CuBi_2_O_4_ nanofibers’ (**a**,**b**) SEM images and (**c,d**) TEM images; (**e**) the elemental mapping after depositing Pt.

As indicated in Figure S1b (Supplementary Materials), a typical digital photo of the as-spun film before annealing shows white. After high-temperature annealing, the photocathode shown in Figure S1c (Supplementary Materials) changes to a transparent yellow, which is similar to the spray CuBi_2_O_4_ photocathode. The morphology of the CuBi_2_O_4_ nanofibers was evaluated by TEM and SEM measurements. As shown in Figure 2a, the randomly oriented nanofibers inherited the one-dimensional structure, and the nonwoven film, which was composed of nanofibers, showed typically interconnected flyover-like network form. The high-magnification image of Figure 2b shows these nanofibers possess a porous fiber structure, which has sufficient surface active sites for photocatalytic reaction. As shown in Figure S3 (Supplementary Materials), the average diameter of the porous nanofiber was 225 nanometers, and its length was up to several dozen micrometers. Under low magnification, Figure 2c reveals one-dimensional morphology, and numerous connective nanoparticles make up the products, similar to the SEM results. The distance between adjacent lattice planes was measured to be 0.31 nm, which belongs to the crystal plane (211) of the CuBi_2_O_4_ tetragonal phase (JCPDS 01-080-0996). Figure 2e shows the elemental mapping of the CuBi_2_O_4_ nanofiber after depositing Pt. It can be seen that the Bi, Cu, and O elements were uniformly distributed inside the nanofiber. In addition, the Pt element was uniformly distributed on the surface of the nanofiber.

**Figure 3:**
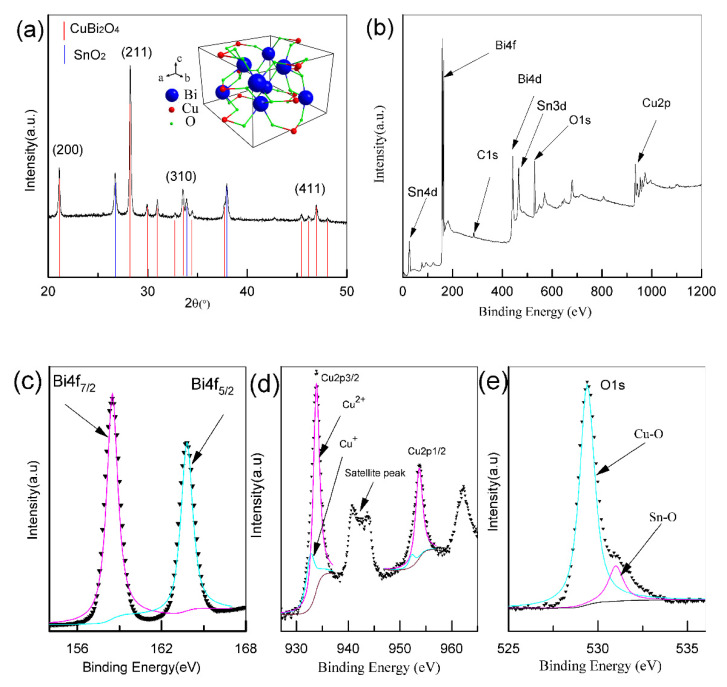
XRD pattern (**a**); XPS spectrum (**b**); high-resolution XPS spectra for (**c**) Bi4f; (**d**) Cu2p; and (**e**) O1s.

As shown in Figure 3a, the diffraction peaks in 20.9°, 28.1°, 33.5°, and 46.7° corresponded to the crystal planes (200), (211), (310), and (411) of the tetragonal CuBi_2_O_4_, respectively (JCPDS 01-080-0996). The SnO_2_ phase of FTO glass was also marked on the same figure with an orange line. No other diffraction peak was found in the XRD pattern, which confirmed the high crystalline and phase purity of CuBi_2_O_4_ after annealing. Detailed information about the surface element composition as well as the chemical state can be obtained by XPS. The survey scan spectrum (Figure 3b) revealed that these nanofibers were composed of Cu, Bi, and O elements. As shown in the high-resolution XPS spectra (Figure 3c–e), two main asymmetric peaks at 159.0 eV and 164.7 eV were attributed to Bi4f_7/2_ and Bi4f_5/2_, corresponding to the oxidation state of Bi^3+^. Then, the peaks at 954.0 and 934.4 eV were attributed to Cu 2p1/2 and Cu 2p3/2. Together with a satellite peak at 942.0 eV, the copper mainly existed in the form of Cu^2+^. Besides, the asymmetrical O1s peak (Figure 3e) ranging from 527 eV to 535 eV was fitted into two peaks at 529.4 and 531.0 eV, which were attributed to Cu–O and Sn–O (SnO_2_ phase of FTO glass) bonds, respectively. Together, both the XRD and XPS results confirmed that these nanofibers were highly crystallized.

**Figure 4:**
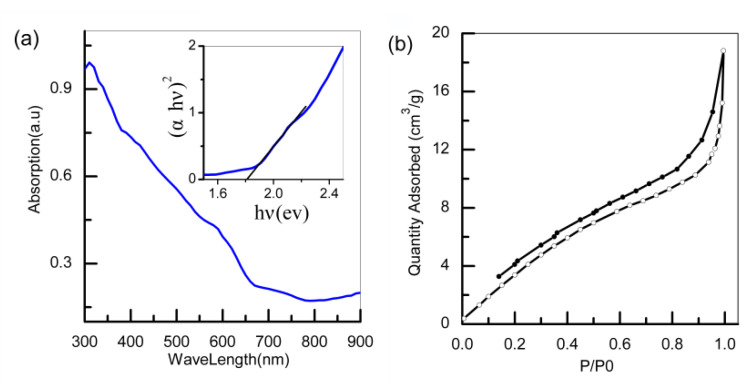
UV-vis absorption spectra (**a**); N2 adsorption–desorption isotherms (**b**) of CuBi2O4 nanofibers.

As indicated in Figure 4a, the CuBi_2_O_4_ nanofibers exhibited strong UV-vis absorbance in both the ultraviolet and visible light regions, and their absorption cutoff wavelength was about 650 nm. By employing the linear part of (αhv)^2^ vs. hv, the band gap was calculated to be 1.8 eV, which is similar to that reported in other studies (1.74 eV) [17]. From the nitrogen adsorption and desorption isotherms of CuBi_2_O_4_ nanofibers (Figure 4b), the specific surface area of the CuBi_2_O_4_ nanofibers was calculated to be 20.5 m^2^g^−1^ using the Brunauer–Emmet–Teller model, which was larger than the value (14 m^2^g^−1^) of nanoparticles reported in other research [36].

### 3.2. Photoelectrochemical Performance

**Figure 5:**
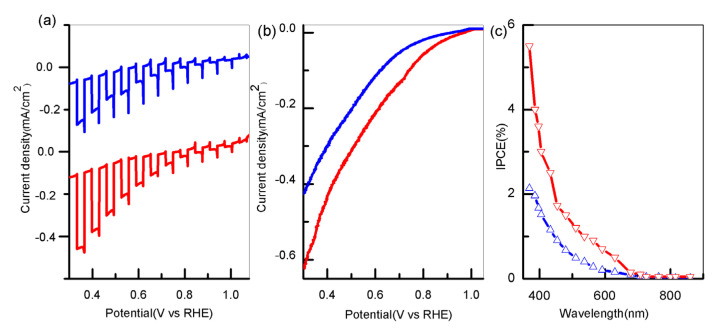
(**a**) Chopped photocurrent vs. voltage curves; (**b**) photocurrent vs. voltage (J–V) curves; (**c**) IPCE spectrum(blue and red lines represent before and after depositing Pt).

To investigate the photoelectrochemical activity of the CuBi_2_O_4_ photocathode, the photocurrent–voltammetry measurement under AM 1.5 was performed. As shown in Figure 5a, the photocurrent started to appear at the initial potential of 1.0 V vs. RHE, increased rapidly when the lamp was turned on, and decreased when the lamp was turned off, indicating that the photocurrent was generated under light irradiation. Interestingly, the instantaneous photocurrent overshoot could also be observed when the lamp was switched on/off, which indicates that electrons accumulate in the space charge layer and reverse recombination occurs between electrons and holes. Moreover, the chopped photocurrent for the CuBi_2_O_4_/Pt nanofibers showed little cathodic transient spikes, which presumably were caused by surface recombination. Figure 5b indicates the photocurrent–voltage (J–V) curves under AM 1.5 irradiation. The photocurrent of CuBi_2_O_4_ nanofibers rose slowly when decreasing the potential, and yielded −0.12 mA/cm^2^ at 0.6 V vs. RHE. On the contrary, after depositing the Pt nanoparticles, their photocurrent rose quickly when decreasing the potential and yielded −0.21 mA/cm^2^ at 0.6 V vs. RHE. Remarkably, the photocurrent of CuBi_2_O_4_/Pt nanofibers was about 75% higher than that of the pristine nanofibers. Then, the IPCE measurement by the tunable light source TLS03 model at 0.6 V vs. RHE was performed. As indicated in Figure 5c, with the increase in illuminant wavelength, the IPCE values gradually decreased to zero at 650 nm (1.8 eV), which was consistent with its band gap energy. Significantly, compared to those of pristine nanofibers (1.8% at 380 nm), the as-prepared CuBi_2_O_4/_Pt nanofibers showed a higher IPCE reaching up to 4% at 380 nm. As shown in Table S1, the nanofibers decorated with Pt exhibit higher photoelectrochemical performance (the value of photocurrent and IPCE) than that of CuBi_2_O_4_ nanofilm (0.15 mA/cm^2^ at 0.6 V vs. RHE) [21]. Nevertheless, the gradient self-doping nanofilm (0.50 mA/cm^2^ at 0.6 V vs. RHE) showed higher photoelectrochemical performance than that of the as-prepared CuBi_2_O_4_/Pt nanofiber due to its internal electric field promoting charge separation. [19].

**Figure 6:**
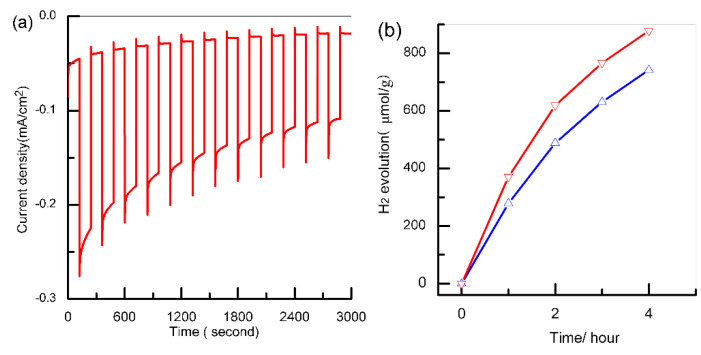
(**a**) Chopped photocurrent vs. time curve at 0.6 V vs. RHE; (**b**) the time courses of H_2_ evolution under AM 1.5 with methanol as a sacrificial reagent (blue and red lines represent before and after depositing Pt).

As indicated in Figure 6a, the photocurrent of the CuBi_2_O_4_/Pt nanofibers photocathode decreased obviously with the increase in time at 0.6 V versus RHE and decreased by nearly 40% after 25 min illumination. Nevertheless, the photostability of the CuBi_2_O_4/_Pt nanofibers photocathode was better than that of the pure Cu_2_O [37] and CuO (52% reduction of photocurrent after 25 min illumination [38]) photocathode. The photocurrent decay was mainly caused by photocorrosion, and a similar phenomenon also appeared at the CuBi_2_O_4_ photocathode consisting of open windows and struts [14]. The photocatalytic H_2_ production of the fabricated nanofibers was measured by the gas chromatography-mass spectrometer (GC-7890B). As shown in Figure 6b, CuBi_2_O_4/_Pt nanofibers exhibited higher photocatalytic performance (380 μmol/(g·h)) compared to the CuBi_2_O_4_ nanofibers (290 μmol/(g·h)). Nonetheless, its photocatalytic performance was much lower than that of Pt/TiO2 nanosheet with exposed (001) facet (8500 μmol/(g·h), [39]). The main reason was that although the one-dimensional nanostructure could reduce the recombination of photogenerated electron–hole pairs, some electron–hole pairs still recombined due to the poor transport property of the carrier (~1.2 × 10^−3^ cm^2^/Vs, [14]). In addition, the photocatalytic activity of these nanofibers slightly decreased with time due to photocorrosion. The instability of CuBi_2_O_4_ nanofibers presents a major challenge for solar water splitting, and protection layers using atomic layer deposition were essential in order to use them as a practical photocathode.

**Figure 7:**
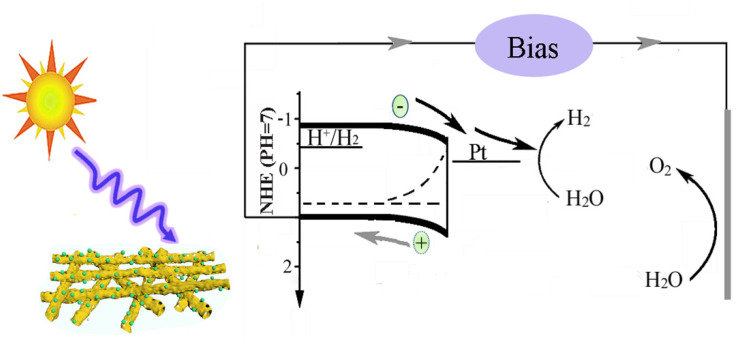
The transport process of electron–hole pairs.

Based on the above analysis of experimental data, the transfer process of the electron–hole pair in the photocathode was shown in Figure 7. Under light illumination, the CuBi_2_O_4_ nanofibers could absorb photons and excite the valence electron to the conduction band. Then the photogenerated electron moved to the interface between the photocathode and the electrolyte due to the downward band bending and injected into the electrolyte to take part in the reduction reaction of hydrogen, which was similar to the charge transfer of BiVO_4_ anode [35]. As shown in Figure 5a, there are amounts of recombination of electron–hole pairs during the water splitting reaction. The photoelectrochemical test demonstrates that the CuBi_2_O_4_/Pt nanofibers show better photocatalytic activity. The main reasons are as follows: Firstly, nanofibers have a large specific surface area and porous structure, which maintain good contact with electrolytes and enrich the active sites. Moreover, the nanofibers, possessing a one-dimensional structure, can also shorten the length of hole diffusing to the FTO substrate and decrease the recombination of the electron–hole pair. In addition, the Pt cocatalyst can also efficiently extract a photogenerated electron from the space charge layer. Therefore, more electrons can be transferred into electrolytes to take part in the reduction reaction of hydrogen, and the electron–hole recombination is significantly hindered. In summary, the main reasons for the enhanced photoelectrochemical performance of CuBi_2_O_4_/Pt nanofiber photocathode are porous nanofibers and cocatalysts.

## 4. Conclusions

In summary, the platinum-enriched porous CuBi_2_O_4_ nanofibers (CuBi_2_O_4_/Pt) with uniform coverage and high surface area were prepared as a photocathode through an electrospinning and electrodeposition process for improving the photoelectrochemical water splitting. The CuBi_2_O_4_ nanofibers showed an average diameter of 225 nanometers, and lengths up to several dozens of micrometers. The porous nanofiber structures allow the cocatalyst (Pt) to have better alignment on the surface of CuBi_2_O_4_, which can effectively hinder the electron–hole recombination at the electrolyte interface. These nanofibers have a tetragonal crystal structure, and their band gap was determined to be 1.8 eV. After depositing Pt nanoparticles, their photocurrent density was 0.21 mA/cm^2^ at 0.6 V vs. RHE under AM 1.5 illumination, and the IPCE was 4% at 380 nm. The enhanced photoelectrochemical ability was mainly attributed to the porous nanofibers, large specific surface area, and the cocatalytic activity of Pt nanoparticles. This work shows a new view for integrating an amount of Pt nanoparticles with CuBi_2_O_4_ nanofibers, indicating the synergistic effect of cocatalysts for efficient storage of solar energy into hydrogen.

## Data Availability

The data presented in this study are available upon request from the corresponding author.

## Supplementary Materials

The following are available online at https://www.mdpi.com/article/10.3390/polym13193341/s1, Figure S1: Digital micrograph of (a) solution precursor, (b) PVP-CuBi_2_O_4_ nanofiber mat before annealing and (c) CuBi2O4 nanofiber mat after annealing; Figure S2: TG-DSC curve of the crystallization of CuBi2O4 nanofiber; Figure S3: CuBi_2_O_4_ nanofiber (a) SEM, (b) corresponding diameter distribution; Table S1: Comparison of photocurrent data reported in the literature with the photocurrent value obtained in the present study.
