# Supplementary material for: An Electrospun Porous CuBi2O4 Nanofiber Photocathode for Efficient Solar Water Splitting
Source: Polymers (Basel). 2021 Sep 29;13(19):3341. doi: 10.3390/polym13193341 (PMC8512590; doi:10.3390/polym13193341)
Supplement: Supplementary file 1 [file polymers-13-03341-s001.zip › polymers-1294298-supplementary.pdf]

Supplementary Materials

An Electrospun Porous $CuBi_2O_4$ Nanofiber Photocathode for Efficient Solar Water Splitting

Xiuhua Yuan [1], Yeling Liu [2], Hui Yuan [2], Bingxin Liu [2], Tianyu Guo [3], Huawei Zhou [2] and Xia Li [2,*]

[1] School of Mechanical and Automotive Engineering, Liaocheng University, 252000 Liaocheng, China; yuanxiuhua@lcu.edu.cn
[2] Department of Chemistry, Liaocheng University, 252000 Liaocheng, China; 1910120301@stu.lcu.edu.cn (Y.L.); 2010120316@stu.lcu.edu.cn (H.Y.); 2018204269@stu.lcu.edu.cn (B.L.); huaweizhou@lcu.edu.cn (H.Z.)
[3] Department of Art and Science, University of Vermont, Burlington, 05405 Vermont , USA; tguo18@jh.edu
* Correspondence: lixia@lcu.edu.cn

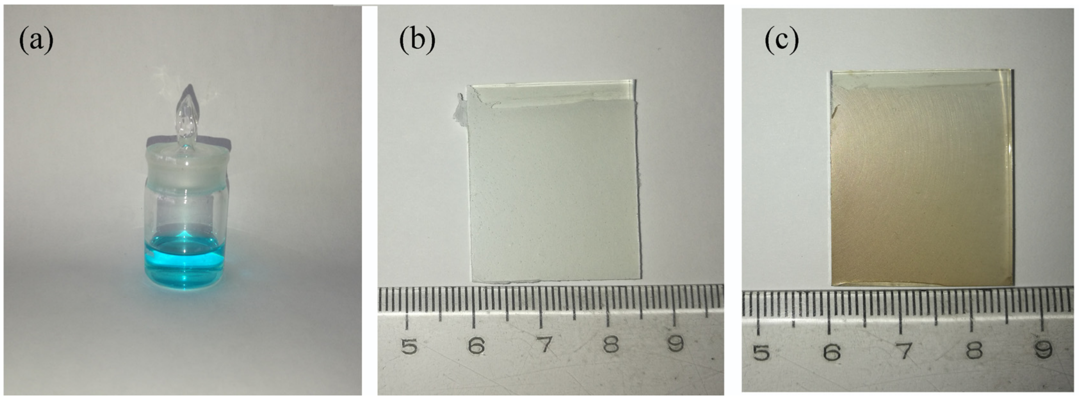

Figure S1. Digital micrograph of (a) solution precursor, (b) PVP-$CuBi_2O_4$ nanofiber mat before annealing and (c) $CuBi_2O_4$ nanofiber mat after annealing

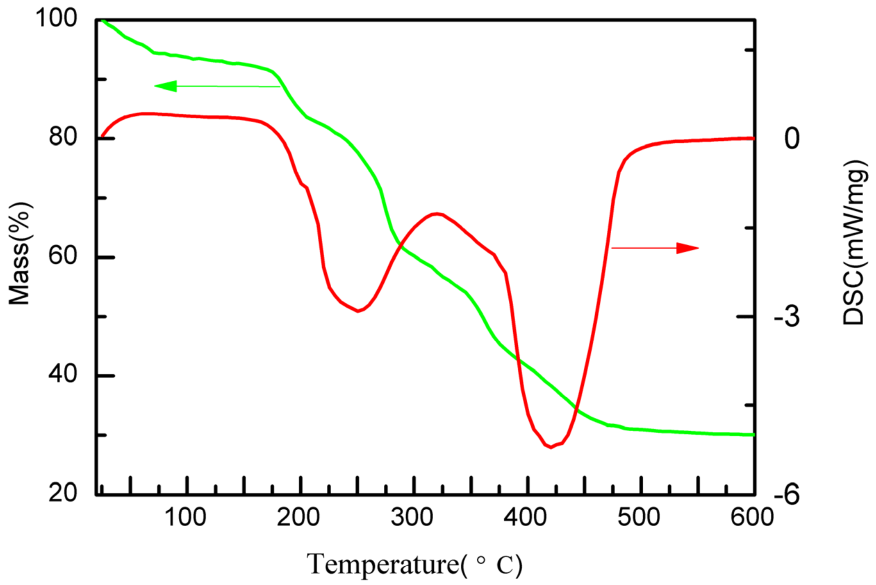

Figure S2. TG-DSC curve of the crystallization of $CuBi_2O_4$ nanofiber

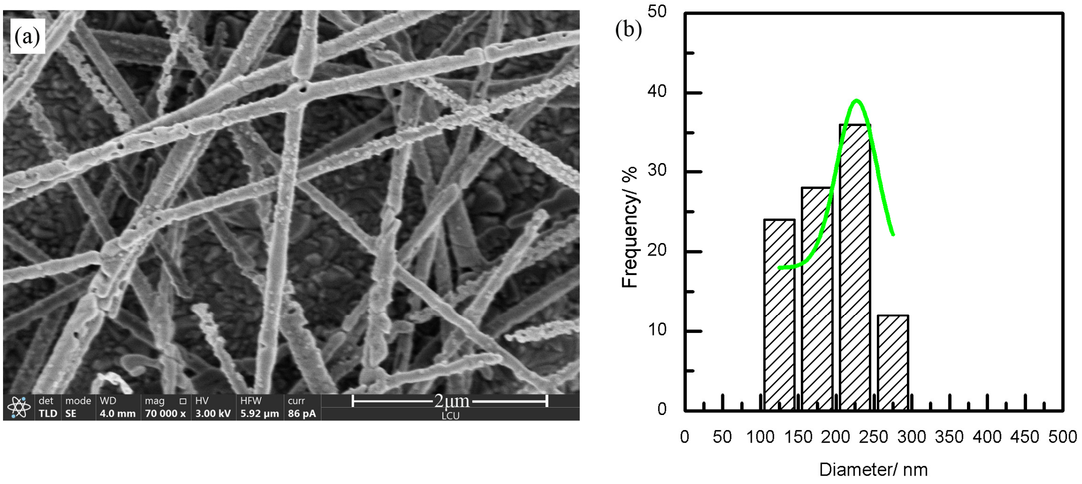

Figure S3. $CuBi_2O_4$ nanofiber (a) SEM, (b) corresponding diameter distribution

Table S1. Comparison of photocurrent data reported in the literature with the photocurrent value obtained in the present study

Fabrication method	Photocathode	Band gap(eV)	Performance (at 0.6 V RHE)	Ref
Pulsed laser deposition	pristine nanofilm	1.92	0.02 mA/cm^2	1
electrochemical deposition	nanoparticle film	1.6	0.06 mA/cm^2	2
Spray pyrolysis	pristine nanofilm	1.46	<0.30 mA/cm^2	3
drop casting	nanotextured films	1.82	0.09 mA/cm^2	4
drop casting	porous films with Cu:Bi=0.55	1.67	0.14 mA/cm^2	5
drop casting	nanofilm consist of open windows and struts	1.5	0.15 mA/cm^2	6
**Electrospun**	**pristine nanofiber**	**1.8**	**0.12 mA/cm^2**	**This work**
drop-casting	nanofilm with gradient self-doping	/	0.50 mA/cm^2	7
**Electrospun**	**nanofiber decorated with Pt**	**1.8**	**0.21 mA/cm^2**	**This work**

Reference

1. Gottesman, R.; Song, A.; Levine, I.; Krause, M.; Chemseddine, A. Pure $CuBi_2O_4$ photoelectrodes with increased stability by rapid thermal processing of Bi_2O_3/CuO grown by pulsed laser deposition. Adv. Funct. Mater. 2020, 30, 1910832- 1910845.

2. Cao, D.; Nasori, N.; Wang, Z.; Yan, M.; Yong, L. p-type $CuBi_2O_4$: an easily accessible photocathodic material for high-efficient water splitting. J. Mater. Chem. A 2016, 4, 8995-9001.

3. Wang, F.; Chemseddine, A.; Abdei, F.; Krol, R.V.D.; Berglund, S.P. Spray pyrolysis of $CuBi_2O_4$ photocathodes: improved solution chemistry for highly homogeneous thin films. J. Mater. Chem. A 2017, 5, 12838 - 12847.

4. Li, J.; Griep, M.; Choi, Y. S.; Chu, D. Photoelectrochemical overall water splitting with textured $CuBi_2O_4$ as a photocathode. Chem. Commun. 2018; 54, 3331-3334.

5. Xu, Y.; Jian, J.; Li, F.; Liu, W.; Jia, L.; Wang, H. Porous $CuBi_2O_4$ photocathodes with rationally engineered morphology and composition towards high-efficiency photoelectrochemical performance. J. Mater. Chem. A. 2019, 7, 21997-22004.

6. Berglund, S. P.; Abdi, F. F.; Bogdanoff, P.; Chemseddine, A.; Friedrich, D.; Roel V.D.K. Comprehensive evaluation of $CuBi_2O_4$ as a photocathode material for photoelectrochemical water splitting. Chem. Mater. 2016, 28, 4231–4242.

7. Wang, F.; Septina, W.; Chemseddine, A.; Abdi, F.F.; Friedrich, D.; Bogdanoff, P.; Krol, R.V.D.; Tilley, D.; Berglund S.P. Gradient self-doped $CuBi_2O_4$ with highly improved charge separation efficiency. J. Am. Chem. Soc. 2017, 139, 15094 - 15103.
